# Pharmacokinetic Analyses of Liposomal and Non-Liposomal Multivitamin/Mineral Formulations

**DOI:** 10.3390/nu15133073

**Published:** 2023-07-07

**Authors:** Joungbo Ko, Choongsung Yoo, Dante Xing, Drew E. Gonzalez, Victoria Jenkins, Broderick Dickerson, Megan Leonard, Kay Nottingham, Jacob Kendra, Ryan Sowinski, Christopher J. Rasmussen, Richard B. Kreider

**Affiliations:** Exercise & Sport Nutrition Lab, Department of Kinesiology and Sport Management, Texas A&M University, College Station, TX 77843, USA; joungboko10@tamu.edu (J.K.); choongsungyoo@tamu.edu (C.Y.); dantexing@tamu.edu (D.X.); dg18@tamu.edu (D.E.G.); victoria.jenkins@tamu.edu (V.J.); dickersobl5@email.tamu.edu (B.D.); meganleonard10@tamu.edu (M.L.); kvnottingham@tamu.edu (K.N.); rjs370@tamu.edu (R.S.); crasmussen@tamu.edu (C.J.R.)

**Keywords:** bioavailability, nutrient absorption, vitamin A, vitamin E, vitamin B_12_, vitamin C, calcium, iron, magnesium

## Abstract

Recent research supports previous contentions that encapsulating vitamins and minerals with liposomes help improve overall bioavailability. This study examined whether ingesting a liposomal multivitamin and mineral supplement (MVM) differentially affects the appearance and/or clearance of vitamins and minerals in the blood compared to a non-liposomal MVM supplement. In a double-blind, randomized, and counterbalanced manner, 34 healthy men and women fasted for 12 h. Then, they ingested a non-liposomal (NL) or liposomal (L) MVM supplement and a standardized snack. Venous blood samples were obtained at 0, 2, 4, and 6 h after MVM ingestion and analyzed for a panel of vitamins and minerals. Plasma levels of vitamins and minerals and mean changes from baseline with 95% confidence intervals (CIs) were analyzed using general linear model statistics with repeated measures. The observed values were also entered into pharmacokinetic analysis software and analyzed through univariate analysis of variance with repeated measure contrasts. The results revealed an overall treatment x time interaction effect among the vitamins and minerals evaluated (*p* = 0.051, $\eta_{p}^{2}$ = 0.054, moderate effect). Differences between treatments were also observed in volume distribution area (vitamin E, iron), median residence time (vitamin E, iron), volume distribution area (iron), volume of distribution steady state (vitamin A, E, iron), clearance rates (vitamin A, E), elimination phase half-life (vitamin E, iron), distribution/absorption phase intercept (vitamin A), and distribution/absorption phase slope and rate (vitamin C, calcium). Vitamin volume distribution was lower with liposomal MVM ingestion than non-liposomal MVM sources, suggesting greater clearance and absorption since similar amounts of vitamins and minerals were ingested. These findings indicate that coating a MVM with liposomes affects individual nutrient pharmacokinetic profiles. Additional research should evaluate how long-term supplementation of liposomal MVM supplements may affect vitamin and mineral status, nutrient function, and/or health outcomes.

## 1. Introduction

Liposomes are spherically shaped vesicles that are created from lipids [1,2,3]. Because of their hydrophilic and hydrophobic characteristics, liposomes have been used to encapsulate drugs and nutrients to promote intestinal absorption, delivery, and bioavailability [1,4]. Encapsulating drugs and/or nutrients with liposomes provides a protective barrier around the compound, thereby increasing resistance to digestive enzymes, acidity, intestinal flora, and oxidation [2]. This helps protect the nutrient from degradation and oxidation as well as protect the digestive tract from potential irritation by the nutrient, thereby improving delivery and bioavailability to target tissues. Liposomal encapsulation helps protect a nutrient or drug from deterioration and can also help target delivery to the target gland, tissue, or system where it can be utilized [4]. While liposomal encapsulation technology has been used to enhance drug delivery to tissues, there has also been interest in use of this technology in nutraceutical applications [5]. For example, liposomal encapsulation has been used to enhance the absorption and delivery of vitamin C [6,7,8,9], folate [10,11], vitamin A [12,13], vitamin D [14,15], vitamin E [15,16,17], calcium [18], and iron [19,20], among other nutrients [12,21,22,23].

Multivitamin and mineral supplements (MVMs) have been consumed for decades and are among the most popular dietary supplements [24]. The American Medical Association [25] and the National Institutes of Health [26] recommend that individuals take a daily multivitamin to ensure the availability of essential nutrients. Additionally, an international panel of nutrition experts performed a Delphi analysis of the available literature and concluded that ingestion of a daily multivitamin might help reduce nutritional deficiencies in susceptible populations [27]. Moreover, the International Society of Sports Nutrition recommends that active individuals and athletes take a multivitamin (with iron for females) to help meet micronutrient needs, particularly during heavy training periods [28]. For this reason, ingestion of a daily multivitamin is a widespread practice to promote general health.

More recently, there has been interest in determining whether ingesting a liposome coated MVM supplement may enhance vitamin and mineral bioavailability compared to a non-liposomal MVM. For example, Tinsley and colleagues [20] reported that ingesting a MVM supplement encapsulated with liposomes improved iron but not magnesium absorption compared to a ingesting a standard MVM supplement [20]. However, this is the only study we are aware of that examined the effects of coating a MVM supplement with liposomes on the bioavailability of vitamins or minerals contained in a MVM supplement. Good scientific practice is to conduct at least two studies from independent labs to determine if results are consistent and reproduceable [26,29,30]. For example, in the United States, federal agencies like the Defense Advanced Research Projects Agency (DARPA) have been funding two studies concurrently at independent labs using the same design and methods for several years to provide original and replication data [29]. Moreover, the Federal Trade Commission requires that competent and reliable scientific evidence from a sufficient number of randomized clinical trials, including replication studies from independent labs, be performed to substantiate structure and function claims [31]. Thus, these initial findings need replication and assessment of how this liposomal MVM supplement not only affects the bioavailability of iron and magnesium, but also other vitamins and minerals contained in the MVM supplement.

Given the above, the sponsor of the Tinsley and coworkers study [20] provided a grant to our lab while they were completing their study to independently conduct a second study using the same experimental design, liposomal and non-liposomal supplements, measurement time points, and methods to collect, store, process, and analyze samples to determine if two independent labs would find similar results. However, we not only evaluated the impact of iron and magnesium, but we also assayed a broader array of vitamins and minerals and performed a more comprehensive pharmacokinetic analysis to further explore how coating a MVM supplement with liposomes affects the appearance, absorption, and/or clearance of vitamins and minerals from the blood. Theoretically, if coating a MVM supplement with these liposomes alters the appearance, clearance, and/or pharmacokinetic profile of vitamins and minerals contained in a MVM supplement, it could affect the bioavailability and/or functionality of the nutrients. The primary outcomes were plasma vitamin and mineral changes and calculated pharmacokinetic variables. A secondary outcome was the perception of side effects. We hypothesized that the liposomal MVM would promote greater bioavailability than a non-liposomal MVM.

## 2. Methods

### 2.1. Experimental Design

This study was conducted as a randomized, crossover, double-blind, placebo-controlled study in a university setting. The Human Protection Institutional Review Board (IRB2021-1418F) approved this study in accordance with ethical standards for the conduction of human participant research as described in the Declaration of Helsinki. This clinical trial was registered with the International Standard Randomized Control Number registry (ISRCTN61456591). Multivitamin ingestion served as the independent variable. The primary outcome was serum vitamin levels and area under the curve measures. Comprehensive pharmacokinetic analysis variables served as the secondary outcome. We hypothesized that ingesting a liposomal vitamin and mineral multivitamin formulation would promote a more sustained appearance and elimination of vitamins and minerals than a non-liposomal multivitamin.

### 2.2. Study Participants

**Figure 1:**
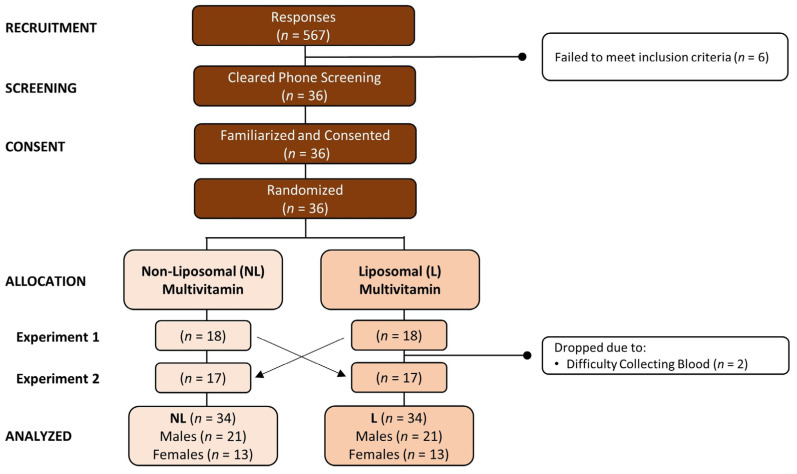
Consolidated standards of reporting trials (CONSORT) diagram for the non-liposomal (NL) and liposomal (L) treatments.

Healthy males and females were recruited to participate in this study. Eligibility criteria included being between 18 and 65 years of age at the time of consent, the ability to comply with study procedures, and availability to complete the study based on durations of individual visits and scheduling requirements. Exclusion criteria included (1) presence of a disease or medical condition that could reasonably influence study outcomes or make participation inadvisable; (2) use of medication that could reasonably influence study outcomes or make participation inadvisable; (3) inability to abstain from medication, supplement, or substance use during the overnight fast and duration of the study visit; (4) anticipated inability to provide blood samples (e.g., known difficulty providing blood samples); and/or (5) currently pregnant or breastfeeding, based on self-report. Figure 1 shows a consolidated standards of reporting trials (CONSORT) diagram. A total of 567 individuals responded to study advertisements and were assessed for eligibility. Of these, 36 passed the phone screening, consented to participate in the study, and were familiarized and randomized into treatments. Treatment assignments are shown by testing rounds with the number of participants evaluated (*n*) displayed. Two participants withdrew from the study due to difficulty collecting blood samples. A total of 34 participants (21 males, 13 females) completed the study and were included in the analysis.

### 2.3. Testing Protocol

**Figure 2:**
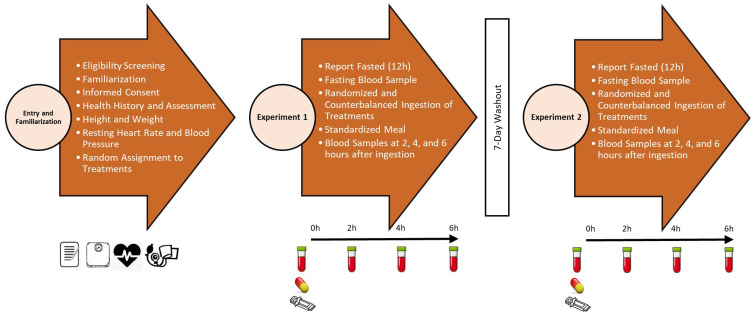
Overview of experiment study timeline.

Figure 2 presents the testing sequence employed in this study. Participants were recruited via email, post, and/or publishing participant flyers in local online and/or print venues. Volunteers expressing interest in participating in the study underwent a phone screening to determine general eligibility. Participants meeting phone eligibility criteria were invited to a familiarization session where they were informed about the study and signed an informed consent statement. Consenting participants then completed a health history questionnaire and underwent a general health screening that included determination of height, weight, resting heart rate, and blood pressure. Those meeting entrance criteria were scheduled for the first experimental testing session. Participants reported to the lab after a 12 h fast from food, dietary supplements, medications, and intake of all substances except water. Participants donated a fasting venous blood sample and then consumed the assigned supplement along with a standardized meal. Blood samples were taken 2, 4, and 6 h after ingestion of the meal and supplement. Participants observed a 7- to 14-day washout period and reported to the lab in a fasted state and repeated the experiment while consuming the remaining assigned treatment.

### 2.4. Liposomal Multivitamin Preparation

**Figure 3:**
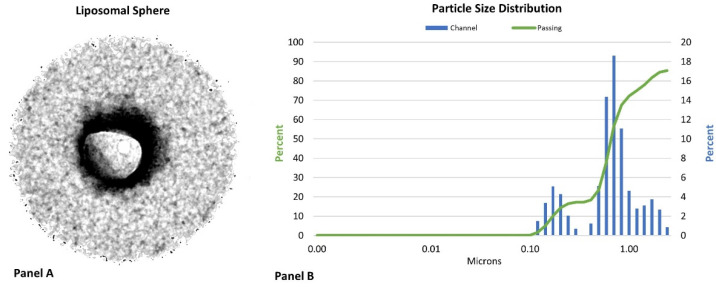
Cryogenic transmission election microscopy (TEM) image of a liposomal sphere created using the encapsulation methods used in this study (Panel (**A**)) and particle size distribution of the liposomal multivitamin and mineral supplement as determined through dynamic light scattering (DLS) analysis (Panel (**B**)). The TEM image was provided courtesy of David M. Belnap, PhD, from the Electron Microscopy Core Laboratory at the University of Utah (Salt Lake City, UT, USA) and the DLS data were provided by Emek Blair, PhD, from CELLg8 labs (Wellington, CO, USA).

Raw materials needed to prepare the liposomal multivitamin and mineral supplements were purchased by the sponsor and converted to liposomes by CELLg8 labs (Wellington, CO, USA) using the methods described by Davis et al. [7] and Tinsley et al. [20]. Briefly, this involved mixing 136 mg of natural sunflower phospholipids with 284 mg multi-vitamin blend under inert conditions in a 304l stainless reaction vessel, allowing the lipids to orient around the payload at room temperature according to the partial charge of the molecules. CELLg8 labs then coated the exterior of a MVM supplement using their proprietary liposomal encapsulation technology. Liposome sphere encapsulation was verified at the Electron Microscopy Core Laboratory at the University of Utah using a cryogenic transmission electron microscopy (TEM) technique [32] with a Tecnai F30 TEM (Field Electron and Ion company, Hillsboro, OR, USA). This essentially involves freezing samples, assessing the negative staining TEM to ensure liposome-like particles are present from viewing the outside of the particles, and then evaluating a cryo-specimen that assesses the inside of the liposomes [32]. Particle size at 158 nm was determined after digestion using dynamic light scattering (DLS) using a Nanotrac Flex analyzer (Microtrac, Verder Scientific, Newton, PA, USA). According to the developers of this liposomal encapsulation technology, this process delivers a thicker layer of liposome spheres surrounding a compound than other labs (see Figure 3, Panel A); over 90% encapsulation efficiency (see Figure 3, Panel B); and improved bioavailability [7,20].

### 2.5. Supplementation Protocol

Supplements were administered in a double-blind, randomized, and crossover manner using a balanced Latin square method to counterbalance the order of treatment administration [33]. Treatments included (1) a non-liposomal multivitamin (Nutraceutical Corp., Salt Lake City, UT, USA) and (2) a liposomal multivitamin (Solaray Liposomal Multivitamin Universal, Salt Lake City, UT, USA) manufactured by Nutraceutical Corp. (Salt Lake City, UT, USA). Supplements were prepared using good manufacturing procedures, assayed, and certified for content by CELL8g Labs and Nutraceutical Corp. Table 1 shows the ingredients of the supplements studied. Supplements were the same in terms of size and appearance and were packaged in generically labeled bottles for double-blind administration by Solaray. Participants ingested the assigned supplement with 8 ounces of water after consuming a standardized snack (Nature Valley Oats’ N Honey crunch granola bar, General Mills, Inc, Minneapolis, MN, USA) in a similar manner to Tinsley et al. [20]. According to the nutrition facts label, two granola bars contained 190 calories, 7 g fat (1 g saturated, 0 g trans fats), 0 g cholesterol, 140 mg sodium, 29 g carbohydrate (2 g dietary fiber, 11 g total sugars, 11 g added sugars), 3 g protein, 12.8 mg calcium, and 1 mg iron. The rationale of co-ingesting a standardized snack with the multivitamin treatments was to promote the intestinal absorption of nutrients by providing macronutrients that influence absorption rates.

## 3. Procedures

### 3.1. Demographics

Weight and height measurements were obtained using a calibrated (±0.02 kg) digital scale (Health-O-Meter Professional 500KL, Pelstar LLC, Alsip, IL, USA). Resting hemodynamics were obtained in the seated position after resting for 5 min. Heart rate was determined via palpation of the radial artery, while resting blood pressure was determined via oscillation of the brachial artery using a stethoscope and mercurial sphygmomanometer according to standard procedures [34].

### 3.2. Blood Collection

Fasting blood was obtained before ingestion of the treatments and ingestion of the standardized breakfast as well as at 2, 4, and 6 h after ingestion of the supplement and snack, following methods described by Tinsley and coworkers [20]. Approximately 25 mL of whole blood was obtained from an antecubital vein in the forearm for each data point using standard phlebotomy procedures [35,36]. Blood was collected in serum separation (SSTs) and Lithium Heparin Vacutte^®^ tubes (Becton, Dickinson and Company, Franklin Lakes, NJ, USA). The SSTs were left at room temperature for 15 min while the lithium heparin tubes were placed in an ice bath and protected from light exposure. Samples were then centrifuged for 10 min at 3000× *g* in a refrigerated (4 °C) Thermo Scientific Heraeus MegaFuge 40R Centrifuge (Thermo Electron North America LLC, West Palm Beach, FL, USA) [37]. Serum was extracted from the SSTs and lithium heparin tubes, placed in several labeled 1 mL micro-storage containers, and stored at −80 °C.

### 3.3. Nutrient Assays

All samples were shipped on dry ice to Heartland Assays LLC (Ames, IA, USA) for analysis. Vitamin A (retinol) and vitamin E (α-tocopherol) were measured in serum as previously described [38] on an Agilent 1100 high-pressure liquid chromatography (HPLC) system (Agilent, Santa Clara, CA, USA) using a C-18 column coupled to an ultraviolet diode array detector (UV-DAD) for quantitation. Vitamin C was analyzed in lithium heparin serum using HPLC [39]. Vitamin B_12_ from serum was analyzed by using liquid chromatography–mass spectrometry (LC/MS/MS; Agilent 1290/6460 Series Triple Quadrupole LC/MS System, Agilent, Santa Clara, CA, USA) [40,41,42,43]. Serum calcium (Pointe Scientific, Canton, MI, USA), magnesium (Pointe Scientific, Canton, MI, USA), and iron (Sigma-Aldrich, St. Louis, MO, USA) were analyzed using colorimetric assay methods as described in the respective methods.

### 3.4. Pharmacokinetic Analysis

The vitamin and mineral dosage, weight of participants, and serum vitamin and mineral values observed for each experiment were entered into the PK Solutions 2.0 pharmacokinetic software using single-dose analysis with 2 terms (Summit Research Services, Montrose, CO, USA). This normalized results by body weight and differences in nutrient dosage between treatments. The software calculates the single-dose elimination phase and disappearance/appearance slope, rate, and half-life as well as concentration max (Cmax), time max (Tmax), area under the curve (AUC), area under the moment curve (AUMC), mean residence time (MRT), volume distribution area (Vd), steady-state volume distribution area (Vss), clearance area (CL), elimination rates, and distribution/absorption rates. Values calculated from each experiment for each treatment were statistically analyzed to determine whether the different multivitamin sources differentially affected pharmacokinetic profiles.

### 3.5. Statistical Analysis

Sample size was determined assuming an expected improvement of 5% with a power of 80% in primary outcome variables. An n-size of 25 was determined to have the necessary power in a crossover design. Participants were randomized to treatments in a crossover manner using a balanced Latin square designer program [33]. Data were analyzed using the IBM^®^ Version 28 SPSS^®^ statistical analysis software (IBM Corp., Armonk, NY, USA). Serum vitamin and mineral levels were analyzed using general linear model (GLM) multivariate and univariate analyses with repeated measures of time and treatments. Sphericity was assessed using Mauchly’s test, while skewness and kurtosis statistics assessed normality. The Wilks’ lambda and Greenhouse–Geisser univariate correction tests were used to assess time and treatment × time interaction effects. Pairwise differences were assessed using Fisher’s least significant difference statistics. We also examined treatment × time × sex effects. The clinical significance of findings was also evaluated by assessing mean changes with 95% confidence intervals (CIs). Means and 95% CIs entirely above or below baseline were considered clinically significant [44]. Chi-square analysis was used to assess time max results. Pharmacokinetic (PK) variables were assessed using univariate analysis of variance (ANOVA) with repeated measures. The probability of type I errors (*p*-level) was set at 0.05 or less. Statistical tendencies were noted when *p*-values were between 0.05 and 0.10. Data are means ± standard deviations (SD) or 95% CIs. Partial eta squared ($\eta_{p}^{2}$) values were used to assess effect size, where values of 0.01 represented a small effect, 0.06 represented a medium effect, and 0.14 represented a large effect size [45].

## 4. Results

### 4.1. Demographic Data

Table S1 shows participant demographic data. Participants were 27.6 ± 7.7 years old and 168.9 ± 9.6 cm tall, weighed 69.9 ± 14.4 kg, had a body mass index (BMI) of 24.3 ± 3.5 kg/m^2^, a resting heart rate of 75.9 ± 9.6 bpm, a systolic blood pressure of 115.6 ± 14.2 mmHg, and a diastolic blood pressure of 73.0 ± 9.1 mmHg, and consumed 448 ± 102 mL of water during the experiment. Sex differences were observed in all demographic parameters except age and the amount of water ingested during the experiments.

### 4.2. Multivariate Analysis

No significant treatment x time x sex differences were observed in plasma vitamin and minerals (*p* = 0.979, $\eta_{p}^{2}$ = 0.017, small effect), unadjusted Cmax, Tmax, or AUC (*p* = 0.998, n_p_^2^ = 0.006, small effect), or detailed PK analysis responses except for weight-related variables. Given this, we only report treatment x time effects. Serum vitamin and mineral results observed among treatments are shown in Table S2a. Multivariate analysis revealed a significant time (*p* < 0.001, $\eta_{p}^{2}$ = 0.383, large effect) and treatment by time effect (*p* = 0.051, $\eta_{p}^{2}$ = 0.054, moderate effect) on vitamin and mineral levels in response to treatment administration, indicating that the pharmacokinetic profiles differed among the vitamins and minerals analyzed. A similar response pattern was observed when expressing data as percentage changes from baseline. The following describes the univariate analysis of changes in individual vitamin and mineral levels as well as pharmacokinetic analysis.

### 4.3. Fat-Soluble Vitamins

#### 4.3.1. Vitamin A

**Figure 4:**
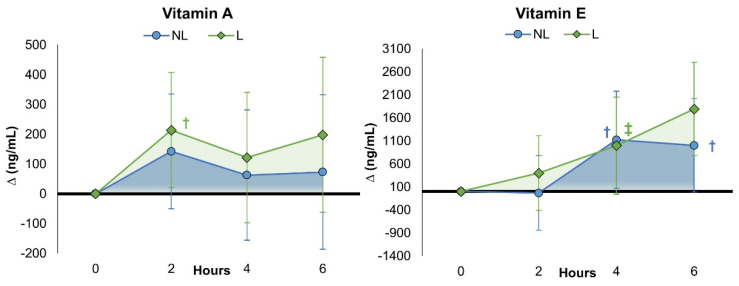
Change in serum fat-soluble vitamins after ingestion of non-liposomal (NL) and liposomal (L) multivitamins. Data are mean changes from baseline with ±95% confidence intervals. † = *p* < 0.05 (‡ = *p* > 0.05 and <0.10) difference from baseline.

As shown in Table S2a, the univariate analysis of vitamin A values revealed no significant time (*p* = 0.217, $\eta_{p}^{2}$ = 0.022, small effect) or time x treatment interaction effects (*p* = 0.897, $\eta_{p}^{2}$ = 0.003, small effect). Vitamin A levels increased significantly from pre-treatment values after 2 h of liposomal MVM ingestion. However, no significant changes from baseline were observed among treatments (see Figure 4). When expressed as percent changes from baseline (Table S2b), vitamin A levels in the NL treatment group were higher than baseline after 2 h, while values with L treatment tended to progressively increase over time, peaking at 6 h. Table S3 presents the PK analysis results. Significant treatment effects were observed among PK variables (*p* < 0.00, $\eta_{p}^{2}$ = 0.739, very large effect). The volume of distribution steady-state (Vss) area tended to be lower (*p* = 0.099, $\eta_{p}^{2}$ = 0.041, moderate effect), meaning that a lower dose can be provided to achieve a given plasma concentration [46], while systemic clearance (observed area) tended to be higher (*p* = 0.061, $\eta_{p}^{2}$ = 0.052, moderate effect) and clearance rate (exponential) was significantly higher (*p* = 0.043, $\eta_{p}^{2}$ = 0.060, moderate effect) with liposomal MVM ingestion.

#### 4.3.2. Vitamin E

Table S2a shows the vitamin E results. Univariate analysis showed that vitamin E levels increased over time (*p* = <0.001, $\eta_{p}^{2}$ = 0.086, medium effect), with no significant difference for treatment effects (*p* = 0.592, $\eta_{p}^{2}$ = 0.009, small effect). Vitamin E levels increased after 4 h with NL while tending to increase with L treatment. After 6 h, vitamin E levels were significantly above baseline levels with L treatment while tending to be higher with NL treatment. However, no significant differences were observed among treatments. When expressed as a percentage change from baseline (Table S2b), there was an upward trend for vitamin E to increase with L treatment at 6 h (NL 11.7% [−0.7, 23.4], L 21.7% [8.9, 32.5], *p* = 0.281), suggesting that absorption into the blood was continuing. Analysis of mean changes from baseline with 95% CIs revealed similar findings. PK analysis (Table S3) found that the volume of distribution area (*p* = 0.078, $\eta_{p}^{2}$ = 0.046, moderate effect) and AUC exponential (*p* = 0.082, $\eta_{p}^{2}$ = 0.045, moderate effect) tended to be lower with L treatment, suggesting a greater clearance rate since dosage in absolute or relative terms were similar. In support of this finding, mean residence time area (*p* = 0.063, $\eta_{p}^{2}$ = 0.052, medium effect), exponential mean residence time (*p* = 0.072, $\eta_{p}^{2}$ = 0.048, medium effect), distribution volume observed area (*p* = 0.051, $\eta_{p}^{2}$ = 0.057, medium effect), and elimination half-life values (*p* = 0.064, $\eta_{p}^{2}$ = 0.051, medium effect) tended to be lower with L treatment, while steady-state volume distribution values tended to be higher (*p* = 0.086, $\eta_{p}^{2}$ = 0.044, medium effect) with L treatment.

### 4.4. Water-Soluble Vitamins

#### 4.4.1. Vitamin B_12_

Table S2a shows the vitamin B_12_ (cobalamin) results. Univariate analysis revealed no significant time (*p* = 0.353, $\eta_{p}^{2}$ = 0.014, small effect) or treatment x time effects (*p* = 0.326, $\eta_{p}^{2}$ = 0.015, small effect). Pairwise comparisons revealed no impact of supplementation on B_12_ levels from baseline or between treatments after 2, 4, and 6 h of ingestion. This was also evident when evaluating delta value changes from baseline with 95% CIs (see Figure 4). However, when expressed as percentage changes from baseline (Table S2b), vitamin B_12_ was only significantly increased above baseline with L treatment at 2 h (NL 31.3% [−80.6, 143.3], L 123.5% [11.6, 235.5], *p* = 0.249). Likewise, no significant treatment effects were observed among PK variables (*p* = 0.747, $\eta_{p}^{2}$ = 0.720, small effect) and no significant differences were observed between treatments for B_12_ PKA values. However, PK data could only be calculated for 33 of 68 experiments. Therefore, the B_12_ PK results must be interpreted with caution.

#### 4.4.2. Vitamin C

**Figure 5:**
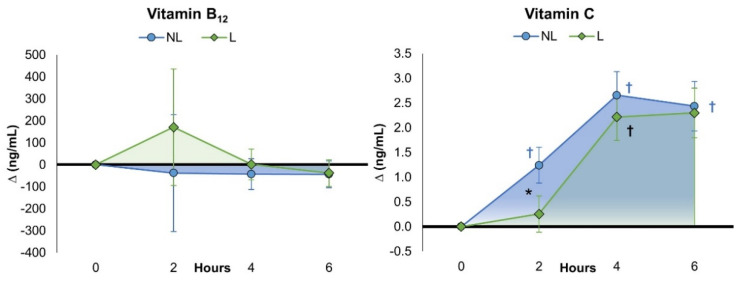
Change in serum water-soluble vitamins after ingestion of non-liposomal (NL) and liposomal (L) multivitamins. Data are mean changes from baseline with ±95% confidence intervals. † = *p* < 0.05 difference from baseline; * = *p* < 0.05 between treatments.

Table S2a shows the vitamin C (ascorbic acid) results. Univariate analysis revealed significant time (*p* = <0.001, $\eta_{p}^{2}$ = 0.632, very large effect) and treatment x time effects (*p* = 0.028, $\eta_{p}^{2}$ = 0.053, medium effect). Post hoc analysis revealed a more delayed increase in vitamin C levels following L treatment, with NL treatment values tending to be higher than L values at hour 2 (NL 8.49 µg/mL [7.2, 9.7], L 6.91 [5.6, 8.2], *p* = 0.088). However, vitamin C levels were significantly increased with all treatments after 4 and 6 h with no differences among treatments. When expressed as a percentage change from baseline (Table S2b), the mean difference at hour 2 was significantly different between treatments (NL 23.3% [11.9, 34.7], L 2.8% [−8.6, 14.2], *p* = 0.013). Differences among treatments were also seen when evaluating mean changes from baseline with 95% CIs (Figure 5). Table S3 shows that treatments tended to differ in vitamin C PK-related variables (*p* = 0.101, $\eta_{p}^{2}$ = 0.532, very large effect). There was evidence that the distribution/absorption phase slope and rate (*p* = 0.082, $\eta_{p}^{2}$ = 0.050, medium effect) were higher with L treatment compared to NL, suggesting that a lower dose of vitamin C is needed to achieve a given serum concentration [46].

### 4.5. Minerals

#### 4.5.1. Calcium

**Figure 6:**
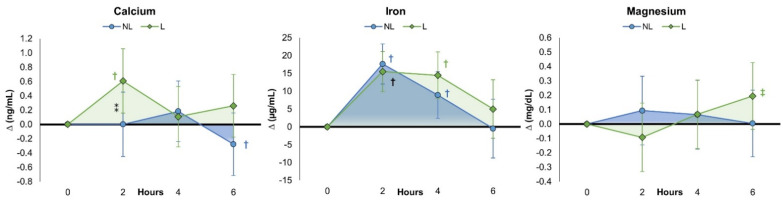
Change in serum mineral levels after ingestion of non-liposomal (NL) and liposomal (L) multivitamins. Data are mean changes from baseline with ±95% confidence intervals. † = *p* < 0.05 (‡ = *p* > 0.05 and <0.10) difference from baseline;⁑ = *p* > 0.05 to *p* < 0.10 difference between treatments.

Table S2a shows changes in calcium levels after oral ingestion of the MVM treatments. Univariate analysis revealed that calcium levels were not changed over time (*p* = 0.220, $\eta_{p}^{2}$ = 0.022, small effect), with no treatments tending to interact (*p* = 0.097, $\eta_{p}^{2}$ = 0.032, small effect). Calcium levels were significantly increased above baseline with L treatment at 2 h, with no statistically significant differences observed between treatments. A similar pattern was observed when expressing data as a percentage change from baseline (Table S2b), with non-significant increases in calcium levels between treatments but with a mean and 95% CI above baseline after 2 h of ingestion in the L treatment (NL 1.6% [−3.3, 6.4], L 6.2% [1.4, 11.1], *p* = 0.183). Analysis of delta changes with 95% CIs revealed that calcium levels increased after L ingestion after 2 h and tended to be significantly greater than NL values at 2 and 6 h (see Figure 6). Table S3 shows that significant treatment effects were observed among PK variables (*p* = 0.053, $\eta_{p}^{2}$ = 0.312, large effect). PK analysis revealed a significant difference between treatments in time to maximum concentration (*p* = 0.032) as well as distribution/absorption phase slope and rate (*p* = 0.024, $\eta_{p}^{2}$ = 0.075, medium effect). Interestingly, although the MVM supplements did not contain calcium, the volume area, mean residence time, and elimination phase variables were lower than those with NL treatment. This finding suggests that the provision of the L MVM may have enhanced the absorption of calcium from the 12.8 mg of calcium contained in the standardized snack and/or pre-existing calcium levels in the blood.

#### 4.5.2. Iron

Table S2a shows serum iron results. Univariate analysis revealed that iron levels increased over time (*p* < 0.001, $\eta_{p}^{2}$ = 0.279, large effect) with no significant treatment x time effects (*p* = 0.208, $\eta_{p}^{2}$ = 0.024, small effect). However, as shown in Table S2b, iron levels only remained significantly above baseline after 6 h with L treatment (NL 6.6% [−9.3, 22.4], L 16.8% [1.0, 32.7], *p* = 0.365), suggesting that the absorption was more prolonged with L treatment. Analysis of mean changes from baseline revealed that iron levels increased 2 and 4 h after ingestion of both MVM treatments, with no significant differences observed between treatments (see Figure 5). PK analysis revealed a significant treatment effect among PK-related variables (*p* < 0.001$,\eta_{p}^{2}$ = 0.732, very large effect). Volume area, mean residence time, volume distribution, and elimination half-life values tended to be lower with L treatment. Since there were no significant differences between treatments in absolute or relative iron doses, these findings suggest greater clearance and absorption of iron with L treatment.

#### 4.5.3. Magnesium

Table S2a presents the observed magnesium values. Univariate analysis revealed that magnesium levels did not change over time (*p* = 0.453, $\eta_{p}^{2}$ = 0.013, small effect), with no significant treatment x time effects (*p* = 0.114, $\eta_{p}^{2}$ = 0.030, small effect). Additionally, no significant differences were observed between treatments in absolute magnesium levels, although values tended to increase above baseline at 6 h with L treatment (see Table S2a). When expressed as a percent change from baseline, magnesium values with L treatment were significantly increased above baseline at 6 h (NL 9.8% [−15.2, 34.9], L 30.8% [5.8, 55.8], *p* = 0.242). These trends are also seen in Figure 6. However, PK analysis revealed no significant treatment effect among PK-related variables (*p* = 0.167$,\eta_{p}^{2}$ = 0.454, very large effect).

### 4.6. Side Effects

Participants did not report any side effects from ingestion of the multivitamin treatments. This observation suggests that the co-ingestion of these multivitamins with a small amount of food was well tolerated.

## 5. Discussion

Encapsulating drugs and/or nutrients with a liposomal layer provides a protective barrier around the compound, thereby increasing resistance to digestive enzymes, acidity, intestinal flora, and/or oxidation. This enhances intestinal absorption, delivery to specific tissues, and/or bioavailability [1,4,5]. While there is evidence that liposomal encapsulation of individual nutrients can affect nutrient absorption into the blood and/or delivery to tissues [1], less is known about whether liposomal encapsulation of a MVM supplement would affect the appearance and/or clearance of vitamins and minerals from the blood. Tinsley and coworkers [20] reported that surrounding a MVM supplement with a liposomal layer enhanced the absorption of iron from the blood with no effect on magnesium. While these findings are interesting, this is also the only study we are aware of that evaluated whether ingesting a MVM coated with a liposomal layer affects the appearance, absorption and/or clearance of vitamins or minerals from the blood, and they only reported the effects on iron and magnesium [20]. It is recommended that at least two independent labs conduct randomized clinical research trails to validate and replicate findings in order confirm results and provide the data necessary to support structure and function claims [26,29,30,31]. Consequently, our group was commissioned by the same sponsor to perform a replication study and more comprehensive pharmacokinetic analysis on a broader array of vitamins and minerals to determine if coating a MVM with this specific liposomal technology affects the absorption and/or clearance of vitamins and minerals from the blood. The results of the present study indicate that ingesting a liposomal MVM supplement alters the overall pattern of the appearance and/or clearance of vitamins and minerals in the blood compared to a non-liposomal MVM. Additionally, there was evidence that ingesting a liposomal MVM affected some pharmacokinetic markers of several individual vitamins and minerals compared to a non-liposomal MVM supplement. These findings do not only provide data to support Tinsley and colleagues’ [20] findings that ingesting a MVM supplement with liposomes may affect the bioavailability of iron; they are novel because we are the first to report on whether coating the outer layer of a MVM with liposomes affects the PK profile of vitamin A, B_12_, C, E, calcium, iron, and magnesium levels collectively and independently. Additionally, we performed a more comprehensive dose- and weight-adjusted PK analysis to account for differences in the vitamin and mineral levels between treatments and compare the effects of equivalent doses relative to body weight on appearance, disappearance/elimination, half-life, and other characteristics. The results support contentions that ingesting vitamins and minerals with a protective liposomal layer influences the rate of appearance, clearance, and/or absorption of nutrients. The following provides additional observations.

### 5.1. Vitamin and Mineral Blood Levels

Before discussing whether differences were observed among different types of MVM supplements, it is important to understand that differences in vitamin and mineral levels in the blood only suggest that absorption rates differ. Higher levels could mean that the source is not taken up as quickly into tissue, while lower levels could mean that less appears in the blood because absorption into tissue is faster [47,48]. Ultimately, target tissues must take up the vitamin or mineral in physiologically meaningful amounts to affect vitamin and mineral status and function. Thus, to fully determine the bioavailability of a nutrient delivery system, it is important to assess differences between arterial (amount delivered to tissue) and venous content (amount remaining in the blood after tissue uptake) as well as to directly determine changes in tissue concentrations over time before conclusions can be drawn. Additionally, to compare the pharmacokinetic profiles of liposomal and non-liposomal MVM supplements in relation to the volume area, residence time, distribution volume, clearance, absorption, and half-life after single-dose administration should be considered. Nevertheless, the first step is to compare whether administration of similar amounts of vitamins and minerals in a liposomal and non-liposomal MVM differentially affects blood concentrations and/or pharmacokinetic variables.

With that in mind, analysis of vitamin and mineral content in the blood after ingestion of the MVM supplements clearly indicated that ingesting a liposomal MVM supplement can affect the rate of appearance and/or clearance of some vitamins and minerals. In this regard, there was an overall interaction effect between the treatments, indicating that coating a MVM with liposomes altered the normal pharmacokinetic profiles. We believe this is the first study to show an overall impact of ingesting a liposomal MVM supplement on the blood levels of a number of vitamins and minerals. However, we also observed differences between treatments in the rate and/or magnitude of increase above baseline values over time in vitamins A, E, and C as well as calcium, iron, and magnesium (see Table S2a,b, and Figure 3, Figure 4 and Figure 5). These findings are consistent with reports that ingestion of liposomal sources of vitamin D [14,15], folate [11,49,50], vitamin C [6,7,18], and iron [20,51] can affect the rate of appearance and/or clearance of vitamins and minerals from the blood. For example, Lukawski et al. [6] reported ingestion of 10 g of sodium ascorbate in liposomal capsules significantly increased blood vitamin C concentrations over 6 h compared to ingesting a standard solid form of vitamin C, as well as demonstrating greater bioavailability in cell culture tests. Davis and coworkers [7] reported that oral administration of 4000 mg of liposomal vitamin C promoted higher plasma vitamin C levels than a non-liposomal form of vitamin C during a 4 h assessment. While the changes observed were significantly less than those for intravenous administration, the researchers concluded that oral administration of liposomal vitamin C was more bioavailable. Moreover, Joseph et al. [18] reported that engineering surface liposomal particles of calcium ascorbate with fenugreek galactomannan enhanced the oral bioavailability of ingesting 1000 mg of vitamin C by about seven times. In the present study, ingesting a liposomal MVM containing about 110 mg of vitamin C promoted a more delayed release of vitamin C over time than ingesting a non-liposomal MVM containing about the same amount of vitamin C. While this is a much smaller dose of vitamin C than has previously been studied, the results demonstrate that ingesting a liposomal MVM supplement affected the time course of release of vitamin C.

The results also support recent findings from Tinsley and colleagues [20], who reported that ingesting a liposomal MVM supplement containing about 9 mg of iron (ferrous glycinate) promoted a more extensive and prolonged increase in blood iron levels from baseline compared to a non-liposomal MVM containing a similar amount of iron. In the present study, ingesting about 9 mg of iron (as glycinate) in a liposomal MVM supplement promoted a more prolonged and consistent increase in iron from baseline than consuming a non-liposomal MVM containing similar amounts of iron. Conversely, Tinsley et al. [20] reported that oral ingestion of a liposomal MVM containing about 22 mg of magnesium (as glycinate) did not differentially affect blood magnesium levels or area under the curve values. In the present study, we found that ingesting a liposomal MVM supplement containing 22 mg of magnesium glycinate promoted a more sustained increase in serum magnesium levels over the 6 h. Additionally, there was evidence that coating a MVM supplement with liposomes increased the absorption of calcium into the blood during the first two hours after ingestion. Collectively, these findings are novel because they demonstrate that coating the outer surface of a MVM with liposomes altered the appearance and/or clearance of several vitamins and minerals from the blood rather than just iron levels.

### 5.2. Dose-Adjusted Pharmacokinetic Analysis

In addition to assessing changes in blood vitamin and mineral levels over time in response to ingesting different types of MVM supplements, this study evaluated pharmacokinetic responses normalized to body weight and dose. This additional analysis is important to account for differences between treatments in the amount of vitamins and minerals consumed as well as to normalize differences in body weight among participants in this study. This analysis also provides more analysis of the area, median residence time, volume distribution, clearance rates, elimination, and absorption pharmacokinetics than typically reported when only assessing changes in blood concentrations in a pharmacokinetic study. When similar doses of a nutrient or drug are consumed, lower values represent greater absorption and/or that less of a dose is needed to reach target tissues [46]. The results of this study indicate that coating a MVM supplement with liposomes can influence the volume distribution and clearance rates of some of the vitamins and minerals contained in the MVM supplement. In this regard, differences among treatments were observed in volume distribution area (vitamin E, iron), median residence time (vitamin E, iron), volume distribution area (iron), volume of distribution steady state (vitamin A, E, iron), clearance rates (vitamin A, E), elimination phase half-life (vitamin E, iron), distribution/absorption phase intercept (vitamin A), and distribution/absorption phase slope and rate (vitamin C, calcium). Vitamin volume distribution was generally lower with liposomal MVM ingestion compared to a non-liposomal MVM source, suggesting greater clearance and absorption since similar amounts of vitamins and minerals were ingested [46]. To date, we are not aware of any other study that has performed this advanced pharmacokinetic analysis on individual nutrients coated with liposomes or a liposomal MVM supplement. While more research needs to assess the impact of ingesting different types of MVM supplements on tissue uptake and concentrations, these findings support contentions that consuming liposomal MVM supplements can influence the appearance and/or absorption of nutrients.

### 5.3. Limitations and Future Directions

With that said, there are some limitations in this preliminary study. First, we were asked by the sponsor to replicate the study design, methods, and assays from Tinsley and coworkers [20]. Their study only took blood samples before and 2, 4, and 6 h after ingesting a liposomal and non-liposomal MVM supplement. Additional insight would have been obtained if more frequent blood samples had been obtained during the initial two hours of supplementation. Moreover, since differences among treatments were observed at the 6 h data point, a longer pharmacokinetic analysis may have also provided additional insight. Second, there were small doses of some vitamins and minerals in the liposomal MVM studied. It is possible that dosage may affect the liposomal delivery and/or protection of some nutrients (e.g., doses larger than 200 mg of vitamin C). Third, co-ingestion of nutrients is known to affect the absorption and clearance of other nutrients as some may have synergistic or inhibitory effects on other nutrients. Therefore, it is unclear how ingesting the different amounts of individual nutrients together may have affected the PK profiles (with or without coating with liposomes). Additional research should evaluate the impact of ingesting MVM supplements with higher amounts of vitamins and minerals. Third, this study examined coating the entire MVM supplement in a liposomal layer. It is unclear whether nano-encapsulation of individual nutrients with liposomes within a MVM supplement may further influence the appearance and/or clearance of specific vitamins and minerals and whether the dosage of individual nutrients may influence bioavailability. Fourth, while some differences were observed in blood concentrations and pharmacokinetics, this initial analysis does not provide insight into whether differences were due to greater cellular or tissue uptake and/or urinary or fecal excretion. Thus, it remains to be determined whether long-term ingestion of liposomal MVM’s may offer any functional and/or health benefits. Finally, statistical trends with moderate to large effect sizes were observed in a number of variables. Consequently, studying a larger sample size may have revealed more consistent statistically significant findings and allowed for additional insight to determine if there were any sex differences. Researchers may want to consider these limitations when planning future work in this area.

## 6. Conclusions

Ingestion of a liposomal MVM supplement differentially affects the concentrations of some vitamins and minerals appearing in the blood, volume distribution, clearance rates, and elimination from the blood compared to a non-liposomal MVM supplement. These findings are important because they are the first to demonstrate that coating a MVM supplement with liposomes can affect the PK profile of several vitamins and minerals within the MVM supplement and thereby influence nutrient bioavailability. With additional research, this may serve as a more efficient way to deliver vitamins and minerals in dietary supplements. However, additional research is needed to determine the impact of coating a MVM supplement with liposomes on tissue uptake, metabolic function, and health. Additionally, it would be interesting to determine whether ingesting individually coated vitamins and minerals with liposomes within a MVM supplement rather than coating the outside of a MVM supplement may yield differential effects on vitamin and mineral bioavailability. Nevertheless, the present findings support contentions that surrounding a MVM supplement with liposomes affects the bioavailability of individual nutrients contained in the MVM supplement.

## Figures and Tables

**Table 1:** Nutrient content of treatments (2-capsule serving).

	Vitamin/Mineral	Unit	Non-Liposomal	Liposomal
Vitamins	Vitamin A (Beta Carotene)	Mcg	950	1098
	Vitamin D_3_	Mcg	22.24	23.90
	Vitamin E (d alpha tocopherol)	Mg	16.38	16.30
	Vitamin K_1_	Mcg	129.00	74.50
	Vitamin B_1_-Thiamine	Mg	0.99	0.91
	Vitamin B_2_-Riboflavin	Mg	0.93	1.10
	Vitamin B_6_-Pyridoxine	Mg	0.97	0.57
	Vitamin B_3_-Niacin	Mg	18.08	17.14
	Vitamin B_5_-Pantothenic Acid	Mg	6.37	5.08
	Vitamin B_7_-Biotin	Mcg	56.78	33.00
	Vitamin B_9_-Folate (5-MTHF)	Mcg	320.60	326.48
	Vitamin B_12_ (Methyl cobalamin)	Mcg	1258	1210
	Vitamin C (Ascorbic Acid)	Mg	108.40	112.70
Minerals	Calcium	%	0.00	0.00
	Chromium (Glycinate)	Mcg	54.72	58.00
	Iodine (Potassium Iodine)	Mcg	171.88	244.00
	Iron (Glycinate)	Mg	10.10	9.38
	Magnesium (Glycinate)	Mg	23.26	22.00
	Manganese (Citrate)	Mg	2.56	2.59
	Molybdenum (Glycinate)	Mcg	54.06	53.60
	Selenium	Mcg	54.70	51.80
	Zinc	Mg	12.82	15.00
Other	CoQ_10_	Mg	4.30	3.78
	Choline (from Bitartrate)	Mg	57.60	44.82
	Inositol	Mg	19.24	22.32
	Lutein	Mg	1.28	1.26
	Para-Aminobenzoic Acid (PABA)	Mg	4.30	4.07

## Data Availability

Data and statistical analyses are available upon request on a case-by-case basis for non-commercial scientific inquiry and/or educational use if IRB restrictions and research agreement terms are not violated.

## Supplementary Materials

The following are available online at https://www.mdpi.com/article/10.3390/nu15133073/s1, Table S1: Demographic data, Table S2a: Vitamins and minerals observed, Table S2b: Percent change from baseline in vitamin and mineral values observed, Table S3: Vitamin and mineral pharmacokinetic data.

## References

[1] Subramani T., Ganapathyswamy H. (2020). An overview of liposomal nano-encapsulation techniques and its applications in food and nutraceutical. J. Food Sci. Technol..

[2] Akbarzadeh A., Rezaei-Sadabady R., Davaran S., Joo S.W., Zarghami N., Hanifehpour Y., Samiei M., Kouhi M., Nejati-Koshki K. (2013). Liposome: Classification, preparation, and applications. Nanoscale Res. Lett..

[3] Al Asmari A.K., Ullah Z., Tariq M., Fatani A. (2016). Preparation, characterization, and in vivo evaluation of intranasally administered liposomal formulation of donepezil. Drug Des. Dev. Ther..

[4] Panahi Y., Farshbaf M., Mohammadhosseini M., Mirahadi M., Khalilov R., Saghfi S., Akbarzadeh A. (2017). Recent advances on liposomal nanoparticles: Synthesis, characterization and biomedical applications. Artif. Cells Nanomed. Biotechnol..

[5] Daraee H., Etemadi A., Kouhi M., Alimirzalu S., Akbarzadeh A. (2016). Application of liposomes in medicine and drug delivery. Artif. Cells Nanomed. Biotechnol..

[6] Lukawski M., Dalek P., Borowik T., Forys A., Langner M., Witkiewicz W., Przybylo M. (2020). New oral liposomal vitamin C formulation: Properties and bioavailability. J. Liposome Res..

[7] Davis J.L., Paris H.L., Beals J.W., Binns S.E., Giordano G.R., Scalzo R.L., Schweder M.M., Blair E., Bell C. (2016). Liposomal-encapsulated Ascorbic Acid: Influence on Vitamin C Bioavailability and Capacity to Protect Against Ischemia-Reperfusion Injury. Nutr. Metab. Insights.

[8] Gopi S., Balakrishnan P. (2021). Evaluation and clinical comparison studies on liposomal and non-liposomal ascorbic acid (vitamin C) and their enhanced bioavailability. J. Liposome Res..

[9] Wen C.J., Chiang C.F., Lee C.S., Lin Y.H., Tsai J.S. (2022). Double Nutri (Liposomal Encapsulation) Enhances Bioavailability of Vitamin C and Extends Its Half-Life in Plasma. J. Biomed. Nanotechnol..

[10] Anderson K.E., Eliot L.A., Stevenson B.R., Rogers J.A. (2001). Formulation and evaluation of a folic acid receptor-targeted oral vancomycin liposomal dosage form. Pharm. Res..

[11] Ling S.S., Yuen K.H., Magosso E., Barker S.A. (2009). Oral bioavailability enhancement of a hydrophilic drug delivered via folic acid-coupled liposomes in rats. J. Pharm. Pharmacol..

[12] Jalali-Jivan M., Rostamabadi H., Assadpour E., Tomas M., Capanoglu E., Alizadeh-Sani M., Kharazmi M.S., Jafari S.M. (2022). Recent progresses in the delivery of beta-carotene: From nano/microencapsulation to bioaccessibility. Adv. Colloid. Interface Sci..

[13] Rovoli M., Pappas I., Lalas S., Gortzi O., Kontopidis G. (2019). In vitro and in vivo assessment of vitamin A encapsulation in a liposome-protein delivery system. J. Liposome Res..

[14] Dalek P., Drabik D., Wolczanska H., Forys A., Jagas M., Jedruchniewicz N., Przybylo M., Witkiewicz W., Langner M. (2022). Bioavailability by design—Vitamin D3 liposomal delivery vehicles. Nanomedicine.

[15] Nowak J.K., Sobkowiak P., Drzymala-Czyz S., Krzyzanowska-Jankowska P., Sapiejka E., Skorupa W., Pogorzelski A., Nowicka A., Wojsyk-Banaszak I., Kurek S. (2021). Fat-Soluble Vitamin Supplementation Using Liposomes, Cyclodextrins, or Medium-Chain Triglycerides in Cystic Fibrosis: A Randomized Controlled Trial. Nutrients.

[16] Cheng C.Y., Barro L., Tsai S.T., Feng T.W., Wu X.Y., Chao C.W., Yu R.S., Chin T.Y., Hsieh M.F. (2021). Epigallocatechin-3-Gallate-Loaded Liposomes Favor Anti-Inflammation of Microglia Cells and Promote Neuroprotection. Int. J. Mol. Sci..

[17] Kala S.G., Chinni S. (2022). Bioavailability enhancement of vitamin E TPGS liposomes of nintedanib esylate: Formulation optimization, cytotoxicity and pharmacokinetic studies. Drug Deliv. Transl. Res..

[18] Joseph A., Kumar D., Balakrishnan A., Shanmughan P., Maliakel B., Im K. (2021). Surface-engineered liposomal particles of calcium ascorbate with fenugreek galactomannan enhanced the oral bioavailability of ascorbic acid: A randomized, double-blinded, 3-sequence, crossover study. RSC Adv..

[19] Ahmad A., Vaghasiya K., Kumar A., Alam P., Raza S.S., Verma R.K., Khan R. (2021). Enema based therapy using liposomal formulation of low molecular weight heparin for treatment of active ulcerative colitis: New adjunct therapeutic opportunity. Mater. Sci. Eng. C Mater. Biol. Appl..

[20] Tinsley G.M., Harty P.S., Stratton M.T., Siedler M.R., Rodriguez C. (2022). Liposomal Mineral Absorption: A Randomized Crossover Trial. Nutrients.

[21] Shariare M.H., Pinky N.J.K., Abedin J., Kazi M., Aldughaim M.S., Uddin M.N. (2022). Liposomal Drug Delivery of Blumea lacera Leaf Extract: In-Vivo Hepatoprotective Effects. Nanomaterials.

[22] Zhang J., Li X., Huang L. (2020). Anticancer activities of phytoconstituents and their liposomal targeting strategies against tumor cells and the microenvironment. Adv. Drug Deliv. Rev..

[23] Dutta S., Moses J.A., Anandharamakrishnan C. (2018). Encapsulation of Nutraceutical Ingredients in Liposomes and Their Potential for Cancer Treatment. Nutr. Cancer.

[24] AlSawaftah N., Pitt W.G., Husseini G.A. (2021). Dual-Targeting and Stimuli-Triggered Liposomal Drug Delivery in Cancer Treatment. ACS Pharmacol. Transl. Sci..

[25] Force U.S.P.S.T., Mangione C.M., Barry M.J., Nicholson W.K., Cabana M., Chelmow D., Coker T.R., Davis E.M., Donahue K.E., Doubeni C.A. (2022). Vitamin, Mineral, and Multivitamin Supplementation to Prevent Cardiovascular Disease and Cancer: US Preventive Services Task Force Recommendation Statement. JAMA.

[26] National Institutes of Health Office of Dietary Supplements Office of Dietary Supplements—Multivitamin/Mineral Supplements. https://ods.od.nih.gov/factsheets/MVMS-Consumer/.

[27] Blumberg J.B., Cena H., Barr S.I., Biesalski H.K., Dagach R.U., Delaney B., Frei B., Moreno Gonzalez M.I., Hwalla N., Lategan-Potgieter R. (2018). The Use of Multivitamin/Multimineral Supplements: A Modified Delphi Consensus Panel Report. Clin. Ther..

[28] Kerksick C.M., Wilborn C.D., Roberts M.D., Smith-Ryan A., Kleiner S.M., Jager R., Collins R., Cooke M., Davis J.N., Galvan E. (2018). ISSN exercise & sports nutrition review update: Research & recommendations. J. Int. Soc. Sports Nutr..

[29] Raphael M.P., Sheehan P.E., Vora G.J. (2020). A controlled trial for reproducibility. Nature.

[30] Zwaan R.A., Etz A., Lucas R.E., Donnellan M.B. (2017). Making replication mainstream. Behav. Brain Sci..

[31] FTC (2023). Issues New Guidance on Health-Related Claims to Replace the Dietary Supplements Advertising Guide. Covington Alert.

[32] Lattin J.R., Belnap D.M., Pitt W.G. (2012). Formation of eLiposomes as a drug delivery vehicle. Colloids Surf. B Biointerfaces.

[33] Kim B., Stein H. (2009). A spreadsheet program for making a balanced Latin Square design. Rev. Colomb. de Cienc. Pecu..

[34] Ferguson B. (2014). ACSM’s Guidelines for Exercise Testing and Prescription 9th Ed. 2014. J. Can. Chiropr. Assoc..

[35] Bowling J.L., Katayev A. (2010). An evaluation of the Roche Cobas c 111. Lab. Med..

[36] Jenkins D.J., Wolever T.M., Taylor R.H., Barker H., Fielden H., Baldwin J.M., Bowling A.C., Newman H.C., Jenkins A.L., Goff D.V. (1981). Glycemic index of foods: A physiological basis for carbohydrate exchange. Am. J. Clin. Nutr..

[37] Bush V.J., Janu M.R., Bathur F., Wells A., Dasgupta A. (2001). Comparison of BD Vacutainer SST Plus Tubes with BD SST II Plus Tubes for common analytes. Clin. Chim. Acta.

[38] Khan A., Khan M.I., Iqbal Z., Shah Y., Ahmad L., Watson D.G. (2010). An optimized and validated RP-HPLC/UV detection method for simultaneous determination of all-trans-retinol (vitamin A) and alpha-tocopherol (vitamin E) in human serum: Comparison of different particulate reversed-phase HPLC columns. J. Chromatography. B Anal. Technol. Biomed. Life Sci..

[39] Karlsen A., Blomhoff R., Gundersen T.E. (2005). High-throughput analysis of vitamin C in human plasma with the use of HPLC with monolithic column and UV-detection. J. Chromatography. B Anal. Technol. Biomed. Life Sci..

[40] Wagner C.L., Shary J.R., Nietert P.J., Wahlquist A.E., Ebeling M.D., Hollis B.W. (2019). Bioequivalence Studies of Vitamin D Gummies and Tablets in Healthy Adults: Results of a Cross-Over Study. Nutrients.

[41] Thayer M.T., Nelssen J.L., Langemeier A.J., Morton J.M., Gonzalez J.M., Kruger S.R., Ou Z., Makowski A.J., Bergstrom J.R. (2019). The effects of maternal dietary supplementation of cholecalciferol (vitamin D(3)) and 25(OH)D(3) on sow and progeny performance. Transl. Anim. Sci..

[42] Zayed A., Bustami R., Alabsi W., El-Elimat T. (2018). Development and Validation of a Rapid High-Performance Liquid Chromatography⁻Tandem Mass Spectrometric Method for Determination of Folic Acid in Human Plasma. Pharmaceuticals.

[43] Frick L.D.R. LC/MS/MS Quantitative Analysis of Water Soluble Vitamins in Blood. Proceedings of the Mass Spectrometry: Applications to the Clinical Lab.

[44] Page P. (2014). Beyond statistical significance: Clinical interpretation of rehabilitation research literature. Int. J. Sports Phys. Ther..

[45] Cohen J. (1988). Statistical Power Analysis for the Social Sciences.

[46] Mansoor A., Mahabadi N. (2022). Volume of Distribution. StatPearls.

[47] Jager R., Purpura M., Shao A., Inoue T., Kreider R.B. (2011). Analysis of the efficacy, safety, and regulatory status of novel forms of creatine. Amino Acids.

[48] Kreider R.B., Jager R., Purpura M. (2022). Bioavailability, Efficacy, Safety, and Regulatory Status of Creatine and Related Compounds: A Critical Review. Nutrients.

[49] Agrawal A.K., Harde H., Thanki K., Jain S. (2014). Improved stability and antidiabetic potential of insulin containing folic acid functionalized polymer stabilized multilayered liposomes following oral administration. Biomacromolecules.

[50] Jampilek J., Kralova K. (2020). Potential of Nanonutraceuticals in Increasing Immunity. Nanomaterials.

[51] Zimmermann M.B. (2004). The potential of encapsulated iron compounds in food fortification: A review. Int. J. Vitam. Nutr. Res..

